# Self vs. Other in Affective Forecasting: The Role of Psychological Distance and Decision from Experience

**DOI:** 10.3390/bs14111036

**Published:** 2024-11-04

**Authors:** Rachel Barkan

**Affiliations:** Guilford Glazer Faculty of Management, Ben-Gurion University of the Negev, P.O. Box 653, Beer-Sheva 8410501, Israel; barkanr@bgu.ac.il

**Keywords:** self–other differences, affective forecasting, psychological distance, decision from experience, utility functions

## Abstract

This work tests self–other differences in the impact bias using the perspectives of psychological distance and decision from description vs. experience. Two studies compared the bias participants made for themselves and for others in a sequential gambling task. The task involved two identical gambles where the first gamble was mandatory and participants made decisions (accept or reject) for the second gamble. Planned decisions were made anticipating a gain or loss in the first gamble, and revised decisions were made following the actual experience of gain or loss. Study 1 compared decisions for self, abstract other, and a close friend. Study 2 replicated the comparison between the self and a close friend and added a measure of empathy. Both studies demonstrated an impact bias indicating that participants tended to overestimate the impact of anticipated outcomes on their tendency towards risk. Specifically, revised decisions indicated risk-aversion shifts after experienced gain and risk-seeking shifts after experienced loss. A reversed pattern emerged for close friends, indicating risky shifts after gain and cautious shifts after loss in Study 1 and for highly empathetic participants in Study 2. Assessing the utility functions that underlie participants’ decisions revealed a qualitative difference. The utility function for the self was consistent with prospect theory (with moderate intensity and diminishing sensitivity), while the utility function for others was more intense with little or no diminishing sensitivity. This research offers new insights regarding the roles of psychological distance and description vs. experience in affective forecasting and impact bias for self vs. other.

## 1. Introduction

Years ago, a pharmaceutical representative approached me with a request to speak to oncologists treating kidney cancer. Typically, I decline consultation talks, but her story piqued my interest. The standard treatment protocol involved administering drug A for 10 consecutive days, followed by a two-month rest period, repeated three times. In contrast, she was promoting drug B, which required continuous 30-day treatment without breaks. Both treatments were equally effective, but patients preferred the drug B protocol, stating that condensing their suffering into a single period is better than stretching it out over several cycles. The doctors were unwilling to adopt the alternative protocol, insisting that extending the patients’ suffering without any breaks was inhumane.

Affective forecasting is the process by which people predict their (and others’) emotional response to future states. Research on affective forecasting (e.g., [1,2,3,4,5]) reveals that while people generally understand the direction of their future feelings, they often misjudge the intensity and duration of these emotions. This miscalculation is known as impact bias, where individuals tend to overestimate how strongly and for how long they will feel certain emotions in response to future events (for comprehensive reviews, see [1,2,3]).

### 1.1. A Brief Description of the Impact Bias

The impact bias has been documented in various groups—students, voters, professors, sports fans, and medical patients—and across numerous contexts such as romantic breakups, receiving negative feedback, decisions about tenure, sports outcomes, electoral results, changes in housing locations, and responses to gains and losses. These studies utilized a range of methodologies, including between- and within-subject designs, one-shot assessments, and longitudinal studies, measuring various emotional and behavioral responses and studying individual differences (e.g., [6,7,8,9,10,11,12,13,14,15,16,17,18,19,20]).

Two primary drivers of impact bias are focalism and immune neglect [1,3]. Focalism refers to the tendency to concentrate on the central aspect of a future event while neglecting other relevant factors and the impact of unrelated general factors. For example, when imagining winning the lottery, people focus on the newfound freedom but ignore potential hassles like tax issues and requests for money from family and friends. Similarly, when contemplating a serious illness, individuals focus on the anticipated fear and suffering, overlooking potential benefits of treatment, social support, and unrelated positive factors like a fulfilling career. Immune neglect involves underestimating our psychological resilience and ability to adapt. For instance, the initial excitement of winning the lottery fades over time, and the fear associated with serious illness is often mitigated by daily coping mechanisms and a return to a sense of normalcy.

### 1.2. Potential Self–Other Differences in the Impact Bias

This work examines the differences between the impact bias we make for ourselves versus the impact bias we make for others. Some research suggests that this difference should not be substantial due to projection bias [19,21,22] that leads people to rely heavily on their own experiences and responses when they consider other people. However, studies on self–other differences show considerable gaps in the way we perceive ourselves compared to how we think about others. For instance, we tend to think of others as more rational [23], more daring [24], and better at handling social rejection [25].

While much research on affective forecasting focuses on errors people make when predicting their own future feelings and responses, there are notable examples of affective forecasts made for others. A famous study by Schkade and Kahneman [5] found that Midwestern students predicted that their fellow Californians would be much happier due to the better climate. In reality, however, there was no difference in self-reported well-being between the two groups. Kolata [26] reported that many healthy Americans stated they did not want to die in a nursing home or hospital. However, 90% of dying patients, most of whom were in acute care hospitals, preferred that option over being cared for by their families. Igou [27] proposed that people overestimate the severity and duration of negative outcomes for others due to an information gap. That is, we understand more about ourselves and our coping mechanisms than we do about others. In line with this rationale, Igou demonstrated that the bias is reduced for familiar people [27]. Recently, however, research found that people worried more about their significant other engaging in risky behaviors that endanger their health than about themselves engaging in those behaviors [28]. Kolata’s report [26] as well as the opening story about the kidney oncologists show how errors in affective forecasting for others can have a significant influence on individual decisions and broader policies.

A critical difference between forecasting for oneself and for others concerns experience. When we forecast for ourselves, we often experience the anticipated event, realize errors in our prediction, and adapt. But when we forecast for others, our predictions remain isolated and cannot be adjusted. At best, we can observe and imagine what the other person is going through (with a risk of further bias).

### 1.3. Applying Two Theoretical Frameworks to the Impact Bias

The current work draws from two theoretical frameworks, applying the main principles of decision from description vs. decision from experience [29,30,31] and construal level theory [32,33] to the impact bias. The two theoretical frameworks offer converging views. The description–experience framework suggests that an impact bias reflects the gap between the response to a description of an anticipated event and the response to the actual experience of that event. Specifically, description is weighed against a neutral reference point, whereas experience shifts our reference points and diminishes our sensitivity to the (subjective) intensity of the event [34,35,36]. Construal level theory views the impact bias as the difference between a high-level representation of an abstract future event and a low-level, concrete representation of that event. Construal level theory further separates the self and the other along a social distance dimension, suggesting that the high-level forecast for a future event would be amplified for the other. Specifically, the forecast for the self refers to a temporally distant event, whereas the forecast for the other refers to a temporally distant event for a socially distant person [32,33,36,37,38,39]. Both perspectives converge, suggesting that the impact bias for the self includes two compensating factors (i.e., description vs. experience or high vs. low construal) whereas the impact bias for the other is isolated without compensation (i.e., only description or only high-level construal).

Thus, the impact bias for the self is driven by (temporal) psychological distance and by actual experience that changes the reference point. The impact bias for others is larger, as it lacks the component of experience on the one hand and increases psychological distance (temporal and social dimensions) on the other hand.

#### 1.3.1. Decision from Description vs. Decision from Experience

**Figure 1:**
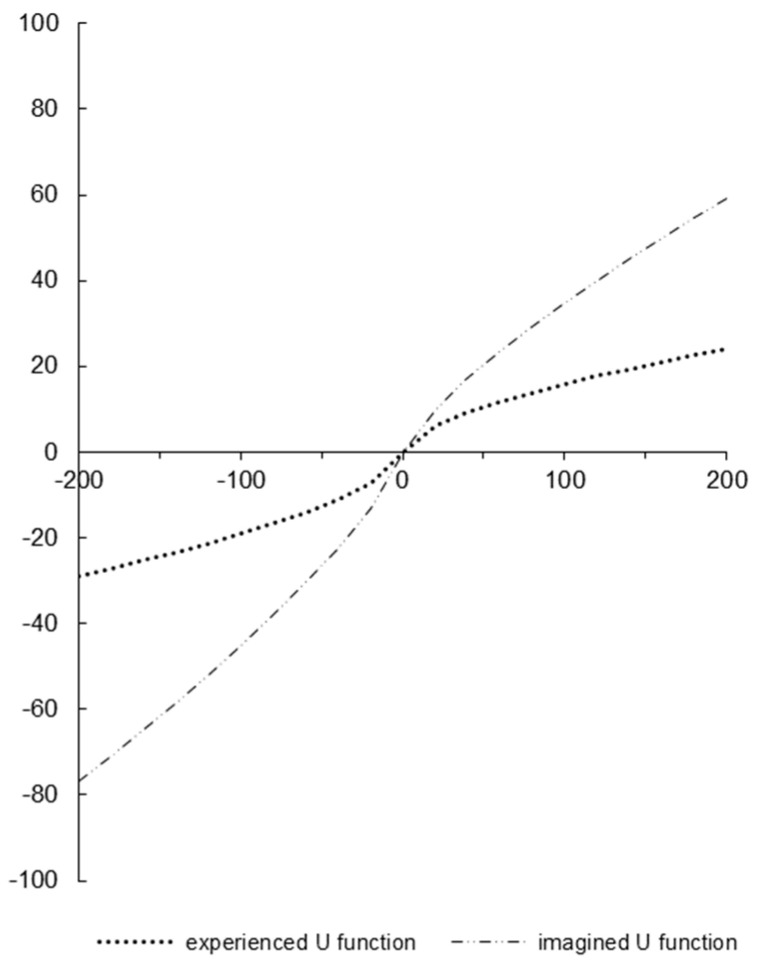
Schematic depiction of experienced and imagined utility functions.

Consider more closely the decision-making perspective of the impact bias as a difference between decision from description and decision from experience (e.g., [29,30,31]). According to this framework, an affective forecast is a short-lived response to a description of a future event. The response to the description is represented as an isolated evaluation that is made against an existing—neutral—baseline. The isolated evaluation highlights the essence of the future event, ignoring anything that might change the context and the re-evaluation of the event (i.e., focalism and immune neglect). When the actual event occurs, two key elements of decision from experience come to play: a shift of the reference point and diminishing sensitivity. A re-evaluation of the event is made against a shifted reference point within the context of diminishing sensitivity, and the event now seems less intense (e.g., [8,10,35]). To exemplify, consider the schematic utility functions in Figure 1. The dotted utility function follows the typical value function of prospect theory [40], highlighting that changes are perceived as more intense when considered in isolation against a reference point of zero (before experience) and less intense when considered against other reference points that are shifted away from neutrality after experience has taken place (e.g., the change from 0 to 100 is more intense than the change from 100 to 200).

The experience-based account that is represented with the dotted utility function has been demonstrated in a sequential gambling paradigm [34,35,36,37,38,39,40,41,42,43,44,45,46]. In this paradigm, participants forecasted their future tendency towards risk (i.e., willingness to accept a gamble) when imagining winning or losing a preceding gamble. Next, after experiencing an actual gain or loss, participants revised their decision (i.e., accept or reject the subsequent gamble). Both forecasts and revised decisions were consequential, and one of them was sampled at random to determine whether the second gamble would be played. The findings demonstrated consistent discrepancies between forecasts and revised decisions that depended systematically on the experienced outcome. Barkan and Busemeyer accounted for the discrepancies with a reference-change mechanism that shifted along a prospect-theory-like utility function [34,35]. The experience of gain tended to shift the reference point ‘up’, leading participants to reject the next gamble that was now perceived as less attractive (i.e., the next possible gain became less intense, and the next possible loss became more intense). The experience of loss tended to shift the reference point ‘down’, leading participants to accept the next gamble that was now perceived more attractive (i.e., the next possible gain became more intense, and the next possible loss became less intense; see the dotted line in Figure 1).

The decision-making perspective emphasizes the reference-change mechanism and diminishing sensitivity. However, these components are more relevant when we consider the impact bias people make for themselves and less relevant when we consider the impact bias people make for others. When forecasting for others, there is no experience, and the process is limited to decision from description. Reference points can still shift as people observe the experience of another person and can imagine how that person would feel. However, without actual experience, there is no diminishing sensitivity and observers cannot adapt. In other words, observing others is limited to decision from description, maintaining focalism and immune neglect, and is therefore expected to result in a stronger impact bias. The dashed line in Figure 1 represents this conjecture with a schematic utility function that lacks diminishing sensitivity and adaptation (i.e., the dashed line is close to linearity). Thus, Figure 1 summarizes the decision-making perspective with two schematic utility functions. An experienced utility function represents the impact bias for the self with a reference-change mechanism. The experienced function allows one to replace focalism and immune neglect with gradual adaptation. An imagined utility function represents a stronger impact bias for the other due to lack of experience and lack of diminished sensitivity.

#### 1.3.2. Construal Level Theory and Psychological Distance

Next, consider the perspective of construal level theory [32,33], adding psychological distance to our understanding of impact bias [37,38,39]. As mentioned above, the impact bias is seen as the gap between a high-level representation of a temporally distant and hypothetical future event and a low-level representation of a proximate, immediate, and actual experience of the event. The high-level construal of a future event is abstract and decontextualized, emphasizing the essence and core intensity of the outcome, lacking details and context. Additionally, the high-level construal does not represent time and changes that may chip off the focal essence. In contrast, the low-level construal of an immediate and actual experience includes concrete details and context [32,33]. Thus, applying construal level theory to the impact bias views focalism as a high-level construal representation where the abstract essence of the future experience is dominant. Immune neglect is viewed as the absence of concrete details and context. The detailed and concrete low-level construal of the actual event opens the door for adaptation (e.g., [32,33,37]).

A potential difference between the impact bias for the self and other is captured with the dimension of social distance. We tend to view others from a distance while reflecting on ourselves more closely [32,33,38,39]. Consequently, it is expected that abstract and decontextualized forecasts for a hypothetical event that is both temporally and socially distant would be amplified for the other as compared to the forecast that is only temporally distant for the self. Specifically, focalism and immune neglect are expected to be stronger when forecasting for others and less intense when forecasting for the self. In line with the perspective of decision-making, the framework of construal level suggests that, considering themselves, people can represent an event as both a high-level construal (i.e., forecast) and low-level construal (i.e., experience). However, since others are distant and since one can only imagine the other’s experience, representation of the event for others can only occur as a high-level construal.

The schematic representation of the imagined and experienced utility functions in Figure 1 is consistent with the suggested conceptualization of construal level theory. Social distance between the self and other is represented with the difference between the two functions. As the self and other are more distant, the different shapes would be more pronounced, and the gap between them should increase. Note that past research implies that social distance could be reduced via information and familiarity, in which case the functions for the self and other should be brought closer [27].

### 1.4. The Two Component Account and Derived Hypotheses

The perspectives of decision from description vs. experience and construal level theory converge and both are represented in Figure 1 with an experienced function for the self and an imagined function for the other. The experienced function emphasizes a *reference-change* mechanism and *diminishing sensitivity*. Focalism and immune neglect are represented with an isolated evaluation of a future outcome against a neutral reference point of zero, creating the forecast. Experience shifts the reference point along the function, and due to diminishing sensitivity, the re-evaluation of the outcome reveals the error of overestimation. The imagined function has little or no diminishing sensitivity. Even if observing another person’s experience shifts the reference point, re-evaluation without diminishing sensitivity would maintain the overestimation of the outcome. In terms of construal level theory, focalism and immune neglect characterize the high-level forecast, whereas the low-level construal of experience is characterized with reference change and diminishing sensitivity. The shift from high-level to low-level construal in the experienced function is sufficient to demonstrate an impact bias for the self. The persistence of high-level construal in the imagined function amplifies the impact bias for the other. As shown in Figure 1, the experienced function that underlies the impact bias for the self is expected to be less intense and the initial slope is expected to gradually flatten. The imagined function that underlies the impact bias for other is expected to be more intense and the steeper slope is expected to endure with little or no flattening.

Two studies test the following hypotheses:

**H1:**  *Impact bias and social distance:* Participants will exhibit an impact bias and forecasting error and the error will be sensitive to social distance (smaller for the self and larger for the other).

**H2:**  *Impact bias and outcome:* The impact bias will indicate systematic changes between planned and revised decisions that are based on a shift of the reference point, and the shift will depend on the outcome (gain or loss).

**H3:**  *Social distance and intensity:* The utility functions that underlie the impact bias will be less intense for the self and more intense for the other (i.e., the slope will be more pronounced for the imagined function as compared to the experienced function).

**H4:**  *Experience and diminishing sensitivity:* The underlying utility functions will exhibit diminishing sensitivity for the self but little or no diminishing sensitivity for the other (i.e., the flattening of the experienced function will be more pronounced as compared to the imagined function).

Two studies utilize the sequential gambling paradigm [34,35,36] to examine the impact bias participants make for themselves and for others. In the first study, social distance is manipulated, asking participants to make forecasts and revised decisions for themselves, for an abstract other, or for a close friend. A second study replicates the comparison between the self and a close friend and adds a measure of empathy using the Interpersonal Reactivity Index (IRI) [41,42] to corroborate and extend the manipulation of social distance.

## 2. Study 1

Study 1 manipulates social distance and compares the impact bias for the self, an abstract other, and a close friend. The impact bias is measured utilizing the sequential gambling paradigm, in which participants make forecasts of their willingness to accept a gamble when anticipating a gain or a loss in a preceding gamble. Next, following the actual experience of a gain or a loss, participants make revised decisions (see details below). According to above hypotheses, participants are expected to exhibit an impact bias. Overestimation of anticipated gains and anticipated losses will be reflected with the difference between planned and revised decisions. The difference should be smallest for themselves and larger for an abstract other. The difference between planned and revised decisions for a close friend is expected to be somewhere in between.

The reference-change mechanism would be demonstrated with consistent changes between planned and revised decisions that systematically depend on the outcome. Finally, the underlying utility functions will differ for self and other, reflecting experienced and imagined functions, respectively. To test the four hypotheses, common statistical analyses (for H1 and H2) are accompanied by assessments of the utility functions (for H3 and H4).

### 2.1. Method

#### 2.1.1. Participants

A total of 120 students from a major college in Israel participated in the study. The participants studied towards the first degree (B.A.). To minimize bias, demographics of age and gender were not collected as there were no specific hypotheses regarding these variables. Israel’s Central Bureau of Statistics report stable demographics over the years 2006–2023 (*M_age* = 25, SD = 5.8 for first year students, *M_women* = 0.60, *SD* = 0.24) [43]. Participants received a show-up fee of NIS 20 and completed the sequential gambling task. Participants received a bonus based on their performance in the experimental task, ranging from NIS 10 to 40.

#### 2.1.2. The Sequential Gambling Task

The sequential gambling paradigm included 16 decision problems. Each problem consisted of two identical gambles with a 50% chance of winning or losing points. The first gamble in each problem was mandatory. The participants’ decision was whether to accept or reject an identical second gamble. Before the first gamble was played, participants made two plans: one for an anticipated gain (“if I win the first gamble, I will accept/reject the second gamble”) and one for an anticipated loss (“if I lose the first gamble, I will accept/reject the second gamble”). After the first gamble was played and the actual outcome was experienced, participants made a revised decision (“I accept/reject the second gamble”). One of the two decisions (the relevant plan for the actual outcome, and the revised decision) was randomly selected to determine whether the second gamble would be played.

Sixteen gambles were used, with expected values (EVs) ranging from −10 points to 50 points in 10-point increments. Each EV was represented by at least two decision problems. For example, for EV = 20, one decision problem was based on a gamble offering a 50% chance to win 140 points or lose 100 points. Another decision problem for EV = 20 was based on a gamble offering a 50% chance to win 200 points or lose 160 points. The outcome of the first gamble was controlled so that participants experienced 8 wins and 8 losses. The outcome of the second gamble was determined randomly. The order of the 16 gambles was counterbalanced. Table 1 presents the set of the 16 gambles, their order of appearance, and their outcome when played for the first time.

#### 2.1.3. The Social Distance Manipulation

The social distance manipulation was presented during the sequential gambling task, when participants were directed to make their planned decisions and when they were directed to make the revised decisions. For planned decisions, the text in the self condition read, “Consider a situation where the 1st gamble is won [lost]. Imagine *yourself* in this situation and reflect on how *you* would feel about winning [losing] the first gamble. Now make two decisions: will *you* accept or reject the 2nd gamble if *you* win the 1st one? Will *you* accept or reject the 2nd gamble if you lose the 1st one?”. In the two other conditions, the text read, “Consider a situation where the 1st gamble is won [lost]. Imagine *a regular student* [*a close friend*] in this situation and reflect on how *they* would feel about winning [losing] the first gamble. Now, make two decisions *through the eyes of that student* [*your close friend*]: will *he or she* accept or reject the 2nd gamble if *they* win the 1st one? Will *he or she* accept or reject the 2nd gamble if *they* lose the 1st one?”.

For revised decisions, the text read “*You* have a chance to revise *your* decision. Do *you* wish to accept or reject the second gamble?” or “The *student* [*your close friend*] has a chance to revise *his or her* decision. Do you think *he or she* will accept or reject the second gamble?”.

#### 2.1.4. Procedure

The study was conducted in a computer class with private cubicles, with each session including 5–7 participants and lasting around 40 min. Upon arrival, participants were seated in their cubicle and read and signed a paper consent form. They then received instructions for the sequential gambling task. The instructions emphasized that both the plans (forecasts) and revised decisions were consequential, with one of them selected at random to determine whether the second gamble would be played. Participants were informed that each point in any gamble equaled NIS 0.10, and that at the end of the study, one of the 16 decision problems would be sampled at random to determine the bonus according to the points that were earned. In practice, only decision problems that ended with positive earnings were sampled to ensure participants did not lose money.

Each trial consisted of four screens displayed sequentially:

**Screen 1** presented the two identical gambles for that trial. Participants were informed that the first gamble was mandatory and were asked to mark their plans for an anticipated gain or loss in that gamble.

**Screen 2** displayed a lottery animation that ended with a pop-up text: “You won [lost] the first gamble. You now have ___ points”.

**Screen 3** required participants to revise their decisions and mark whether they choose to accept or reject the second gamble. A pop-up message then informed participants which decision was sampled (planned or revised) and whether the second gamble would be played.

**Screen 4** was conditioned on whether the second gamble was played. If the second gamble was not played, participants received a summary of the points earned in that decision problem and were instructed to continue to the next decision problem. If the second gamble was played, participants saw another lottery animation and received a pop-up text regarding the outcome of the second gamble as well as the total earnings for the decision problem. “You won [lost] the second gamble. You now have ___ points for the second gamble. Your total earnings for this decision problem are ___ points.” Participants were then instructed to continue to the next decision problem.

Upon completion of the sixteen decision problems, one problem was sampled at random to determine the bonus. Participants were paid the show-up fee and the bonus and were thanked for their cooperation.

#### 2.1.5. Experimental Design

Study 1 manipulated social distance as a between-subject variable (decisions made through the eyes of self/other/friend). Two variables were manipulated within subjects, including outcome (gain/loss) and experience (planned/revised). The experimental design is summarized as a mixed design of 3 × 2 × 2.

#### 2.1.6. Data Analysis

ANOVA with one between-subject variable (social distance) and two within-subject variables as repeated measures (experience, outcome) was carried out to test H1 and H2. These hypotheses would be supported by interactions between the variables.

Assessing participants’ underlying utility functions utilized the reference-change model [35]. Simulating the model provided functions that fit best the sets of planned and revised decisions for individual participants in the self, other, and friend conditions. The reference-change model has been tested for the sequential gambling paradigm and provided the best fit compared to alternative models including a baseline model (where random shifts are considered instead of reference shifts) and a probability model (where systematic shifts in subjective probabilities are considered instead of reference shifts) [35]. Qualitative comparisons of the shape of the utility functions for self, other, and friend, as well as a non-parametric Mann–Whitney test and proximate z-test for the parameters of the utility functions, were carried out to test H3 and H4.

### 2.2. Results and Discussion

Participants were generally risk-seeking and tended to accept the second gamble. The risky tendency was a bit higher for the self (P_accept = 0.62) than for other and friend (P_accept = 0.57 and 0.56, respectively). The general tendency to accept the second gamble is reasonable as the expected value (EV) of 13 gambles was positive, ranging from 10 to 50 points, and the negative expected value of 3 gambles was only −10 points. The general risk-seeking behavior replicates former findings that were reported with this gambling task [35,36].

Table 2 presents the main findings. For each condition of social distance, the table presents the average proportions of the decisions to accept a second gamble (denoted as P_accept). The left side of the table presents the planned decisions for an anticipated gain and for an anticipated loss in the mandatory gamble. The right side of the table presents the revised decisions that followed the experience of an actual gain or an actual loss. The emerging pattern indicates that experience interacts with both the outcome and the social distance conditions.

The changes between planned and revised decisions indicate that participants mispredicted how gains and losses would affect them. When making plans for themselves, participants’ misprediction was minimal for the effect of a gain. Participants predicted that a gain would make them risk-seeking (P_accept = 0.63), but actual experience of a gain decreased their tendency towards risk (P_accept = 0.56). Note that both planned and revised decisions were on the side of risk seeking. This finding resonates with the house money effect [44]. Making decisions for themselves, participants were also risk-seeking in the case of loss. Their planned decisions were less extreme (*p* = 0.58), but an actual loss pushed them to risk seeking (P_accept = 0.65). This finding is in line with prospect theory [40] and the tendency to break even [44]. While participants’ decisions for themselves were consistently risky (above the neutral point of P_accept = 0.50), the risk tendencies participants demonstrated for an abstract other and for a close friend indicated a clearer shift between risk aversion and risk seeking, albeit in different directions. The mispredictions for an abstract other resemble those of the self—only more pronounced. Considering gains, participants thought that others would be a bit more risk-seeking than themselves (P_accept = 0.68), but then they revised their decisions expecting that an actual gain would lead others below risk neutrality (P_accept = 0.47). Considering a loss, the direction was opposite. Participants thought others would avoid further risk (P_accept = 0.44) but, similar to their own experience, an actual loss led them to expect that others would be risk-seeking (P_accept = 0.65). The pattern for the close friend was reversed. (Research has demonstrated reversals of the impact bias, indicating underestimation of affective response. It was offered that classical overestimation is associated with events that are larger in magnitude and duration, more distanced and less expected. Reversal and underestimation was found for events that are less intense, shorter, proximate and expected. This is not the case here. The outcomes and the experience are identical across the experimental condition. Furthermore, the assessed utility functions suggest that the reversal is intertwined with amplified focalism and immune control [45,46]) Participants thought their friends would be risk-averse after a gain (P_accept = 0.42), but following an actual gain, participants revised their decisions, expecting their friends would be risk-seeking (P_accept = 0.62). For losses, participants thought their friend would be risk-seeking (P_accept = 0.68), but then, after an actual loss, revised their decisions and expected their friends would shift to risk neutrality (P_accept = 0.52).

#### 2.2.1. ANOVA with Between- and Within-Subject Analysis

Statistical analysis was carried out for the logit transformations of individual probabilities (unlike probabilities, the logit transformations are distributed symmetrically). A mixed analysis of variance included social distance as a between-subject variable (self/other/friend) and experience (planned/revised) and outcome (gain/loss) as within-subject variables. The main effect of social distance was significant despite the subtle difference in general risk taking between self, other, and friend [*F*(2, 117) = 62.86, *p* < 0.001, *η*^2^_p_ = 0.52]. The main effect of experience was not significant [*F*(1, 117) = 0.67, *p* = 0.416, *η*^2^_p_ = 0.01]. The main effect of outcome was not significant [*F*(1, 117) = 0.16, *p* = 0.900, *η*^2^_p_ = 0.00]. The main effects were not expected to be significant because the effects of planned and revised decisions were expected to be in opposite directions, as would be the effects of gains and losses. The two-way interaction of social distance X experience did not reach significance [*F*(2, 117) = 2.46, *p* = 0.090, *η*^2^_p_ = 0.04]. The two-way interaction of social distance X outcome was marginally significant [*F*(2, 117) = 3.05, *p* = 0.051, *η*^2^_p_ = 0.05]. The two-way interaction of outcome X experience was significant [*F*(1, 117) = 15.69, *p* < 0.001, *η*^2^_p_ = 0.12]. The three-way interaction of social distance X experience X outcome was significant [*F*(2, 117) = 17.64, *p* < 0.001, *η*^2^_p_ = 0.23].

The statistical analysis provides partial support for H1. While the two-way interaction between social distance and experience did not reach significance, the three-way interaction between social distance, experience, and outcome indicated that the difference between the risk tendencies of planned and revised decisions were lowest for the self (delta between the proportions was −0.05 for gain and 0.12 for loss) and largest for other (delta between the proportions was −0.22 for gain and 0.16 for loss). The differences between planned and revised decisions are not completely consistent with H1. In absolute values, the magnitude of the differences resembles the abstract other; however, the directions are opposite (delta between the proportions was 0.18 for gain and −0.16 for loss). The seeming inconsistency would be solved with the assessment of the underlying utility functions.

The statistical analysis fully supports H2. The two-way interaction between experience and outcome is consistent with the conjecture that experience shifts the reference point. The difference between planned and revised decisions represents an initial evaluation of the second gamble against a neutral reference point and a re-evaluation of the second gamble after the actual experience against a different reference point. The directions of change support the notion that the reference point shifts along a prospect-theory-like utility function (for self and for other). Thus, experience of gain shifts the reference point ‘up’, leading to a decrease in the tendency to accept the second gamble. Experience of loss shifts the reference point ‘down’, leading to an increase in the tendency to accept the second gamble. The opposite directions of changes after gain and loss for the close friend suggest that the reference point is shifted along a utility function that differs from prospect-theory-like function. Assessing the utility functions that underlie planned and revised decisions for the self, other, and close friend addresses this possibility and tests H3 and H4.

#### 2.2.2. Assessing Underlying Utility Functions with the Reference-Change Model [35]

Hypotheses H3 and H4 refer to the utility functions that underlie planned and revised decisions in the three social distance conditions. The utility functions for self, other, and friend were assessed with the reference-change model [35]. Past research has applied the reference-change model to the sequential gambling paradigm and demonstrated it offered the best fit compared to alternative models [35]. The assessment of the underlying utility functions was carried at the individual level, finding the best fit for the 32 decisions (i.e., 16 plans and 16 revisions) for each participant.

Barkan and Busemeyer [35] defined the formal reference-change model as a derivation of decision field theory that quantifies the emergence of preferences and represents them in probabilistic terms (e.g., the probability of accepting the second gamble) [47]. To avoid sidetracking, a conceptual description of the model is given here. The complete model is presented in Appendix A, and the syntax for the simulation can be found at https://osf.io/uh3af/?view_only=aa3e1392c1a342e9bf5c0c9d17f69daf (accessed on 25 October 2024).

The reference-change model quantifies the notion that (i) an initial evaluation of the second gamble is made against a neutral baseline, leading to a planned decision; (ii) next, actual experience of a specific outcome shifts the reference point along a utility function; (iii) a re-evaluation of the second gamble is made against that shifted reference point, leading to a revised decision; and (iv) the direction and the magnitude of the gap between planned and revised decisions depends on the magnitude of the experienced outcome and on the shape of the utility function [35]. Thus, if we knew the shape of the utility function, the values of a gamble, and the actual outcome (gain or loss), we could have predicted the planned and the revised tendencies to accept any second gamble. Conversely, based on the observations of the planned and revised decisions for 16 gambles, the reference-change model allows us to assess the shape of the utility function that offers the best fit to the 32 observed decision points (a conceptual analogy would use observations to estimate regression coefficients).

The definition of the utility function follows Tversky and Kahneman’s definition, given with two equations: an equation for gains (U(*v*) = *v*^α^) and an equation for losses—U(*v*) = λ*v*^α^ [48]. The equations include two parameters, *α* and *λ*. The first parameter—*α*—is an exponent that determines the curvature of the utility function. If 0 < *α* < 1, the function exhibits diminishing sensitivity. Lower values lead to a function that is less intense and flattens quickly, and higher values lead to function that is more intense and flattens more gradually (as in the experienced vs. imagined functions in Figure 1, respectively). If *α* = 1, the function will be linear, and if *α* > 1, the function will grow exponentially. In both cases, the functions would lack diminishing sensitivity. The second parameter—*λ*—reflects loss aversion, suggesting people are more sensitive to losses than to gains. This parameter is applied only for losses and, as it increases, it leads to larger asymmetry between the subjective perception of gains and losses.

In their work, Tversky and Kahneman estimated these parameters at *α* = 0.88 and *λ* = 2.25. However, the reference-change model does not force the utility function to follow the typical shape of prospect-theory-like function. For the current assessments, the range for the exponent was set to 0 < *α* ≤ 2, allowing the utility function to either follow the typical prospect theory value function with different levels of diminishing sensitivity [40], or to be linear (or even exponential) with no diminishing sensitivity. Likewise, the range for the loss aversion coefficient was set to 0 < *λ* ≤ 5, allowing one to represent pronounced loss aversion (i.e., *λ* is greater than 1), no loss aversion (where *λ* would be equal to 1), or even a reversal where there is more sensitivity to gains than to losses (in this case, *λ* would be between 0 and 1). The flexible ranges for the parameters are critical to allow the model to capture focalism and immune neglect (as represented in the imagined function in Figure 1). The reference-change model uses two additional parameters. A third parameter titled *mem* estimates participants’ need for consistency (encouraging them to stick to their plan); 0 < *m* ≤ 1. As this parameter is closer to 1, the difference between the planned and revised decision diminishes. The fourth parameter *θ* is central to decision field theory, defining a threshold for the emergence of preferences, 0 < *θ* ≤ 5, to allow flexibility. Note that for the purpose of the assessment of the utility functions, the focus is set on the parameters *α* and *λ*.

**Figure 2:**
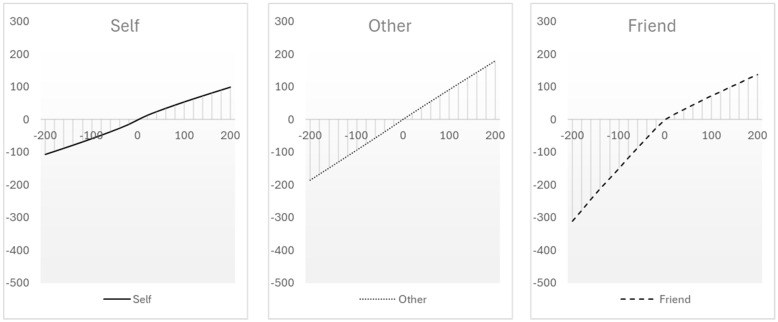
Depiction of median utility functions across social distance conditions.

Table 3 presents the averages, standard deviations, and medians of the two parameters that determine the shape of the utility function for each condition of social distance. Figure 2 depicts the shape of the median utility function for self, abstract other, and close friend.

To avoid assumptions about the distribution of the parameters, median values are preferred as they are less influenced by extreme values. The loss aversion coefficient is similar across the three conditions of social distance, indicating minimal if any overweighting of losses. The difference between the utility functions is driven mainly by the exponent. The utility functions for the self and the abstract other are quite symmetrical for gains and losses, whereas the utility function for the close friend shows some asymmetry (due to the interplay with the exponent being larger than 1).

**Table 3:** Means, standard deviations, and median parameters α and λ across social distance conditions.

	Self		Abstract Other		Close Friend	
	*α*	*λ*	*α*	*λ*	*α*	*λ*
Mean	0.86	1.48	1.10	1.62	1.18	1.42
SD	0.60	1.54	0.59	1.51	0.57	1.14
Median	0.87	1.06	0.98	1.03	1.07	1.07

The utility function for the self resembles a prospect-theory-like shape with moderate intensity and pronounced diminishing sensitivity (median *α* = 0.87). The utility function for an abstract other is more intense, with minimal diminishing sensitivity (median *α* = 0.94, just below linearity). The utility function for a close friend is the most extreme. Surpassing linearity, it depicts the highest level of intensity, no diminishing sensitivity, and an asymmetry that overweigh losses (median α = 1.09).

The Mann–Whitney U test along with the associated Z-test approximation for parameter *α* indicated that the medians for the self and other did not differ significantly [*U* = 644.5, *z* = −1.32, *p* = 0.093]. The medians of parameter *α* for other and friend did not differ significantly either [*U* = 690, *z* = 0.88, *p* = 0.189]. However, the medians of parameter *α* for self and friend differed significantly [*U* = 567, *z* = −2.24, *p* = 0.013]. The findings provide partial support for H3 and H4, indicating that the underlying utility for a close friend (but not for an abstract other) had a more pronounced slope and lower diminishing sensitivity than the utility function for the self. Hypotheses H3 and H4 do not refer specifically to parameter *λ*. The Mann–Whitney U test along with the associated Z-test approximation for parameter *λ* did not indicate any significant differences. The comparisons included self vs. other [*U* = 761.5, *z* = 0.18, *p* = 0.428], other vs. friend [*U* = 701, *z* = 0.77, *p* = 0.220], and self vs. friend [*U* = 764.5, *z* = −0.34, *p* = 0.364]. Thus, the findings do not indicate differences in loss aversion between self, abstract other, and close friend.

The differences between the utility functions are somewhat inconsistent with the expected effects of social distance. According to social distance theory, the most extreme utility function should have been that of the abstract other, who is the most distant from the self. The similarity between the utility functions of the self and the abstract other may suggest that, in the absence of specific information about the abstract other, participants relied more heavily on their own experiences as a basis for the planned and revised decisions regarding the second gamble. This behavior could reflect projection bias and/or empathy gaps [19,21,22]. In contrast, when making decisions for a close friend, with whom participants are more familiar, they may have been more likely to emphasize their friend’s unique characteristics, thus highlighting the differences between their friend and themselves in both planned and revised decisions.

One way to address this possibility is by introducing empathy as a corroborating measure of a related variable that converges with social distance. Empathy includes a cognitive aspect referring to the ability to understand another person’s thoughts from their perspective rather than ours, and an emotional aspect, referring to the ability to vicariously experience another person’s feelings [49,50]. Therefore, high empathy implies psychological proximity, and low empathy implies psychological distance. Study 2 replicated the comparison between the self and close friend, where social distance was demonstrated, and added a measure of empathy. If participants’ familiarity with close friends highlights the differences between their friends and themselves, then low levels of empathy are expected to maintain the distance, and high levels of empathy are expected to reduce that distance.

## 3. Study 2

Study 2 compared the decisions participants make for themselves and for a close friend. An explicit measure of empathy was added, using the Interpersonal Reactivity Index [41,42]. The empathy questionnaire was completed after the sequential gambling task.

### 3.1. Empathy and the Impact Bias

Previous research on affective forecasting that aimed to reduce the impact bias for oneself has demonstrated that people with high levels of emotional intelligence, empathy, and perspective-taking predicted more accurately the intensity and duration of their emotional responses to future events (e.g., [51,52,53]). If empathy has a similar effect on the impact bias people makes for others, then we would expect high empathy to reduce the gap that was found in Study 1 between the self and the close friend. This expectation converges with the expected effect of social distance (see also [27]). On the other hand, some research suggests that empathy works differently when people consider close friends or family members who are significant figures in their lives, suggesting that empathy may increase rather than decrease the impact bias we have for significant others [28].

An additional hypothesis for Study 2 proposes two competing possibilities:

**H5a:**  *Empathy consistency*: If empathy operates in the same way for ‘self’ and ‘friend’ in the context of impact bias, then higher levels of empathy should be associated with smaller discrepancies between planned and revised decisions.

**H5b:**  *Empathy inconsistency*: If, in the context of the impact bias, empathy operates differently for ‘self’ and ‘friend’, then higher levels of empathy should be associated with larger discrepancies between planned and revised decisions.

### 3.2. Method

#### 3.2.1. Participants

A total of 167 students from a major college in Israel participated in the study (84 in a self condition, and 83 in a close other condition). The participants studied towards the first degree (B.A.). To minimize bias, demographics of age and gender were not collected as there were no specific hypotheses regarding these variables. Israel’s Central Bureau of Statistics report stable demographics over the years 2006–2023 (*M_age* = 25, SD = 5.8 for first year students; *M_women* = 0.60, *SD* = 0.24) [43]. Participants received a show-up fee of NIS 20 and completed the sequential gambling task and the IRI questionnaire. Participants received a bonus based on their performance in the sequential gambling task, ranging from NIS 10 to 40.

#### 3.2.2. The Sequential Gambling Task

The sequential gambling task was identical to the one used in Study 1 comparing planned and revised decisions for the self vs. a close friend.

#### 3.2.3. Social Distance Manipulation

Manipulating social distance, the text in the self condition read, “Imagine yourself in this situation and reflect about winning [losing] the first gamble. Now make two decisions: will you accept or reject the 2nd gamble if you win the 1st one? Will you accept or reject the 2nd gamble if you lose the 1st one?”. In the close friend condition, the text read, “Consider a situation where the 1st gamble is won [lost]. Imagine a close friend in this situation reflecting about winning [losing] the first gamble. Now, make two decisions you think your close friend would make: will he or she accept or reject the 2nd gamble if they win the 1st one? Will he or she accept or reject the 2nd gamble if they lose the 1st one?”.

#### 3.2.4. Measuring Empathy with the Interpersonal Reactivity Index

The Interpersonal Reactivity Index [41,42] is a measure of dispositional empathy that conceptualizes empathy as a set of separate but related constructs, including perspective-taking, empathetic concern, fantasy, and personal distress. The questionnaire consists of four seven-item subscales for each construct of empathy. The perspective-taking (PT) subscale measures the reported tendency to spontaneously adopt the psychological point of view of others in everyday life (“I sometimes try to understand my friends better by imagining how things look from their perspective”). The empathic concern (EC) subscale assesses the tendency to experience feelings of sympathy and compassion for unfortunate others (“I often have tender, concerned feelings for people less fortunate than me”). The fantasy (FS) subscale measures the tendency to imaginatively transpose oneself into fictional situations (“When I am reading an interesting story or novel, I imagine how I would feel if the events in the story were happening to me”). The personal distress (PD) subscale taps the tendency to experience distress and discomfort in response to extreme distress in others (“Being in a tense emotional situation scares me”). In Study 2, the questionnaire included three subscales of perspective taking, empathetic concern, and fantasy. The personal distress subscale was considered less relevant [54].

#### 3.2.5. Procedure

The procedure was identical to that used in Study 1. The only difference was that after completing the sequential gambling task, participants completed the empathy questionnaire. Participants were then paid and released, with a thank you for their cooperation.

#### 3.2.6. Experimental Design

Study 2 manipulated social distance between subjects (decisions for self or friend). Two variables were manipulated within subjects, including outcome (gain/loss) and experience (planned/revised decisions). Empathy was measured and later entered as a covariate in the statistical analysis. The experimental design is summarized as a mixed design of 2 × 2 × 2 with a continuous covariate.

#### 3.2.7. Data Analysis

ANOVA with one between-subject variable (social distance), two within-subject variables as repeated measures (experience, outcome), and empathy as a covariate was carried out to test H1 and H2. These hypotheses would be supported by interactions between the variables.

Correlations between empathy scores and the difference in scores between revised and planned decisions were tested separately for the two conditions of social distance (self/friend).

Assessing participants’ underlying utility functions utilized the reference-change model [35]. Simulating the model provided functions that best fit the sets of planned and revised decisions for individual participants in the self, other, and friend conditions. The reference-change model has been tested for the sequential gambling paradigm and provided the best fit compared to alternative models including a baseline model (where random shifts are considered instead of reference shifts) and a probability model (where systematic shifts in subjective probabilities are considered instead of reference shifts) [35]. Qualitative comparisons of the shape of the utility functions for self, other, and friend, as well as a non-parametric Mann–Whitney test and proximate z-test for the parameters of the utility functions, were carried out to test H3 and H4.

### 3.3. Results and Discussion

Empathy scores were computed as the sum of the three components of the questionnaire (perspective taking + empathetic concern + fantasy). Empathy scores for participants who made decisions for themselves ranged from 51 to 100 (*M* = 74.38, *SD* = 9.12, *Med* = 75.50, *N* = 84). For participants who made decisions for a close friend, the empathy scores ranged from 56 to 92 (*M* = 72.63, *SD* = 8.88, *Med* = 72.00, *N* = 83). Participants were generally more risk-seeking and more willing to accept a second gamble when they made decisions for themselves (P_accept = 0.58) and were closer to risk neutrality when they considered a close friend (P_accept = 0.52). Table 4 presents the average proportions of the decisions to accept a second gamble. The left side of the table presents the planned decisions for imagined gain and for an imagined loss in the mandatory gamble. The right side of the table presents the revised decisions that followed the experience of an actual gain or an actual loss. The emerging pattern demonstrates partial replication of the findings of Study 1. When participants made decisions for themselves, the experience of gain created a shift from risk seeking towards risk neutrality, whereas the experience of loss created a shift from risk neutrality towards risk seeking. The average proportions indicate participants were generally risk-neutral when they considered a close friend and did not replicate the reversed shifts that were observed in Study 1.

To demonstrate the effect of empathy, Table 4 also presents the average proportions for subsets of the participants representing the lower quartile of empathy scores (Q1) and the upper quartile of empathy scores (Q4). The proportions for self and for a close friend in the lower quartile (Q1) indicate shifts that are consistent with the general pattern. In the upper quartile (Q4), the shifts for self are consistent with the general pattern, but the shifts for a close friend indicate reversal in the direction of the shifts (i.e., risky shift after gain and cautious shift after loss) indicating a partial replication of the pattern for a close friend in Study 1. The proportions for the lower and upper quartiles of empathy scores are presented for descriptive purposes and not for statistical analysis.

#### 3.3.1. ANOVA with Between- and Within-Subject Analysis with a Covariate

Statistical analysis was carried out for the logit transformations of individual probabilities. A mixed analysis of variance included social distance as a between-subject variable (self/friend) and experience (planned/revised) and outcome (gain/loss) as within-subject variables. Empathy was entered as a covariate in the analysis. The analysis revealed two significant effects. The effect of social distance indicated that participants were significantly more risk-seeking when they made decisions for themselves than for a close friend [*F*(1, 164) = 6.58, *p* = 0.011, *η*^2^_p_ = 0.04]. The three-way interaction of social distance X experience X outcome was significant [*F*(1, 164) = 4.29, *p* = 0.040, *η*^2^_p_ = 0.03]. This three-way interaction supports the pattern that is shown in Table 4 (i.e., the direction of the differences between revised and planned decisions depended on the outcome of gain and loss, and the pattern was pronounced when participants made decisions for themselves, but not when they made decisions for a close friend).

All the other effects were not significant. Empathy did not have a significant effect on the tendency to accept the second gamble [*F*(1, 164) = 0.50, *p* = 0.480, *η*^2^_p_ = 0.00]. The main effect of experience was not significant [*F*(1, 164) = 1.59, *p* = 0.209, *η*^2^_p_ = 0.01]. The main effect of outcome was not significant [*F*(1, 164) = 2.24, *p* = 0.137, *η*^2^_p_ = 0.01]. The two-way interactions were not significant (empathy X experience: *F*(1, 164) = 1.68, *p* = 0.197, *η*^2^_p_ = 0.01; empathy X outcome: *F*(1, 164) = 2.58, *p* = 0.110, *η*^2^_p_ = 0.02). The two-way interactions social distance X experience [*F*(1, 164) = 0.08, *p* = 0.773, *η*^2^_p_ = 0.00], social distance X outcome [*F*(1, 164) = 0.36 *p* = 0.552, *η*^2^_p_ = 0.00], and experience X outcome [*F*(1, 164) = 1.67, *p* = 0.198, *η*^2^_p_ = 0.01] were all non-significant. The three-way interaction of empathy X experience X outcome was also non-significant [*F*(1, 164) = 0.89, *p* = 0.348, *η*^2^_p_ = 0.01].

#### 3.3.2. Correlations Between Empathy Scores and Differences Between Revised and Planned Decisions

Average proportions mask individual differences, and analysis of variance focuses on general tendencies. Going back to Table 4, the proportions presented for the lower and upper quartiles of empathy scores (Q1 vs. Q4) hint that individual differences in empathy may be meaningful. Correlations were computed to uncover the effect of empathy and highlight individual differences. To this end, the raw proportions were replaced with difference scores between the revised and planned decisions for gains and losses. Given the opposite direction of changes following an experience of gain and loss, the correlation between the difference scores was negative. When participants made decisions for themselves, *r*(difference_loss, difference_gain) = −0.75, *p* < 0.001. A similar negative correlation was found when participants considered a close friend: *r*(difference_loss, difference_gain) = −0.70, *p* < 0.001.

In the self condition, where participants made decisions for themselves, empathy scores were not correlated with the difference scores for gains (*r*(empathy, difference_gain) = 0.16, *p* = 0.145) or with the difference scores for losses (*r*(empathy, difference_loss) = 0.03, *p* = 0.811). Thus, when participants made decisions for themselves, empathy was not associated with either smaller or larger forecasting errors. In the friend condition, empathy scores were correlated with the difference scores. Considering a close friend, participants’ empathy score was positively correlated with the difference scores for gains (*r*(empathy, difference_gain) = 0.23, *p* = 0.036). The positive correlation indicates that as participants were more empathetic (i.e., higher scores), they were more likely to make a larger shift away from risk seeking after an experience of gain. A negative correlation was found for losses (*r*(empathy, difference_loss) = −0.23, *p* = 0.034). The negative correlation indicates that as participants were more empathetic (i.e., higher scores), they were more likely to make a larger shift in the opposite direction towards risk seeking. Thus, for both outcomes of gains and losses, higher levels of empathy were associated with larger difference scores—representing larger forecasting errors. The findings in the friend condition support the empathy inconsistency hypothesis H5b.

#### 3.3.3. Assessing Underlying Utility Functions with the Reference-Change Model [35]

**Figure 3:**
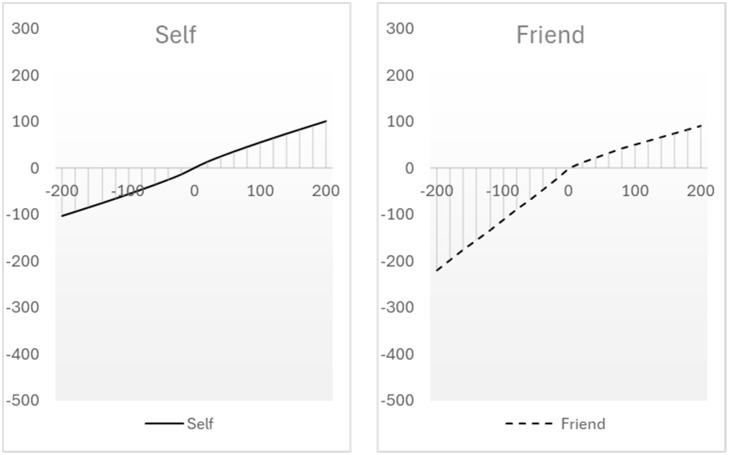
Depiction of median utility functions across conditions.

Utility functions were assessed in the same way as in Study 1. Table 5 presents the averages, standard deviations, and medians of the two parameters that determine the shape of the utility function for the self and for the close friend conditions. These parameters closely resemble those estimated in Study 1, indicating robustness. Figure 3 illustrates the shape of the median utility function for each experimental condition.

As in Study 1, the utility function for the self is less intense, with clear diminishing sensitivity. The utility function for a close friend has little diminishing sensitivity. Unlike Study 1, the utility functions differ further in regard to loss aversion. The utility function for the self is symmetrical for gains and losses, whereas the utility function for a close friend is asymmetrical, indicating that participants were more sensitive when they imagined their friends’ losses than gains.

**Table 5:** Means, standard deviations, and median parameters α and λ across conditions.

	Self		Close Friend	
	*α*	*λ*	*α*	*λ*
Mean	0.97	1.43	1.16	1.77
SD	0.72	1.50	0.65	1.43
Median	0.87	1.02	0.98	1.22

The Mann–Whitney U test along with the associated Z-test approximation for parameter *α* indicated that the medians of parameter *α* were significantly different for self and a close friend [*U* = 2936, *z* = −1.76, *p* = 0.034]. This result replicates the difference that was found in Study 1, providing further support for the hypotheses regarding intensity (H3) and for the hypothesis regarding diminishing sensitivity (H4). Hypotheses H3 and H4 do not refer specifically to parameter *λ*. The Mann–Whitney U test along with the associated Z-test approximation, however, indicated a significant difference between the medians of parameter *λ* for self and a close friend [*U* = 2678, *z* = 2.58, *p* = 0.005].

The asymmetry between losses and gains in the utility function for the close friend implies that participants paid more attention to their friends’ losses than their gains [55], suggesting a higher degree of focalism for friends’ negative experiences. This finding is consistent with past research that defined loss aversion as a forecasting error [8]. Furthermore, this finding provides an elegant demonstration of the theoretical framework of loss attention [55]. According to loss attention, people do not overweight the magnitude of losses over gains, but rather, losses attract more attention than gains. In both studies, participants’ own utility functions were symmetrical. A slight asymmetry in Study 1 and a more pronounced asymmetry in Study 2 suggest that participants did not experience loss aversion when they made decisions for themselves, yet imagined and attributed loss aversion to their close friend [8,55].

## 4. General Discussion

The present work studied the differences in impact bias when individuals make forecasts for themselves versus others. Integrating two theoretical perspectives of psychological distance and decision from description vs. decision from experience provided novel conceptualization and new insights into the impact bias.

Two studies employed the sequential gambling paradigm where participants made forecasts for anticipated outcomes (gain or loss) and revised decisions after experiencing the actual outcome. Study 1 manipulated social distance (self, abstract other, close friend), and Study 2 complemented the manipulation of social distance (self, close friend) with a converging measure of empathy [41,42].

### 4.1. Summary of the Findings

The findings of both studies converged. The findings provided evidence for an impact bias, with an overestimation of the influence of future gain and future loss. An experienced-based reference-change mechanism was evident, demonstrating that changes between revised and planned decisions were systematic and dependent on the experience of the actual outcome. The general pattern demonstrated that experience of gain led to a cautious shift in risk preferences, whereas the experience of loss led to a risky shift in the preferences, replicating past research [34,35,36]. Participants generally made smaller forecasting errors for themselves compared to errors they made for others. The effect of social distance was stronger in Study 1, leading to reversals in the directions of the shifts that followed the experience of gain and loss for a close friend. The effect of social distance was smaller in Study 2, indicating risk neutrality for a close friend (a replication of the reversal that was found in Study 1 emerged for participants with the highest empathy scores).

Empathy did not have a general effect on participants’ decisions. Looking at individual differences, correlations showed that when participants made decisions for a close friend, higher empathy scores were associated with larger (rather than smaller) gaps between revised and planned decisions. This association supported past research indicating that people make larger forecasting errors for significant others [28]. The current findings did not support the notion that empathy reduces the impact bias people make for themselves [51,52,53]. When participants made decisions for themselves, higher empathy scores were not associated with either a decrease or an increase in the gap between revised and planned decisions.

Assessments of the utility functions underlying participants’ decisions indicated further self–other differences. Supporting the integrated view of psychological distance and decision from experience (exemplified in Figure 1), when participants made decisions for themselves, the shape of the underlying utility function was moderate with clear diminishing sensitivity. When participants considered and made decisions for a close friend, the shape of the underlying utility function was more intense (a steeper slope) with little or no diminishing sensitivity.

### 4.2. Contribution to Past Research

This work contributes to the research on impact bias in several ways. First, it highlights how different theoretical perspectives represent and account for the impact bias. The framework of decision-making explains focalism and immune neglect as a decision from description, where future events are considered in isolation without considering actual experience. This perspective extends our understanding and adds that actual experience unravels the impact bias through reference changes, re-evaluations within the context of diminished sensitivity, and inevitable adaptation. The framework of psychological distance represents the impact bias as a discrepancy between the high-level construal of future events and the low-level construal of actual experiences. Focalism and immune neglect emerge from abstract representations of distant events, while low-level representations of actual experiences emphasize concrete details and context, facilitating adaptation. This perspective extends our understanding of the impact bias with the dimension of social distance, providing a basis for distinguishing the impact bias people make for themselves versus the bias they make for others.

Second, utilizing the sequential gambling task presented a novel way to quantify the impact bias and test it in new ways. Employing the reference-change model enabled us to assess the utility functions that underlie the decisions for the self and for others. These utility functions, reflecting participants’ observed decisions, offer a tangible method to present and understand the key drivers of impact bias in a detailed and quantifiable manner.

### 4.3. Limitations and Future Research

The current work has several limitations. One limitation is related to lab experiments. On the one hand, collecting data in the lab provides experimental control. Specifically, the experimenter and the participants were physically present at the same place, and all the participants completed the experimental task from beginning to end in the same uniform way and in the same monitored environment. On the other hand, lab experiments frequently have limited sample sizes. Second, the salience of the MeToo movement inspired the minimal bias approach and specific demographic details of the participants (i.e., gender and age) were not collected as there were no explicit hypotheses regarding the demographics. The latter limitation is compensated by the general information regarding first-year students from Israel’s Central Bureau of Statistics [43]. Third, the data of the current work were collected before the introduction of pre-registration, a priori power analyses. Future research should address these limitations and demonstrate the robustness of the findings using a priori power analyses and larger samples sizes in pre-registered studies. Running the sequential gambling task online via platforms such as MTurk and Prolific is expected to add random noise, and future research may need to find a way to create valid differences between hypotheticality and actual feelings of experience outside the lab. Finally, for aims of generalization, future research should consider the effects of demographics on tendencies towards risk and test them explicitly.

The current work leaves several questions for future research. One question refers to the possibility that the manipulation of social distance [32,33,37,38,39] also elicits a projection bias or empathy gaps [21,22]. Specifically, Study 1 raised the possibility that rather than representing an ‘abstract other’ at the distant high-level construal, participants may have anchored decisions in their own preferences. Future research needs to design an experimental manipulation of psychological distance that avoids the elicitations of the projection bias.

A second question for future research refers to the directions of the shifts between planned and revised decisions in the sequential gambling task. The dominant shifts in the current work and in past research [34,35,36] indicate a cautious shift after the experience of gain and a risky shift after the experience of loss. However, the current findings seem to point at a continuum that goes through risk neutrality (i.e., no systematic directions for a close friend in Study 2) to a reversal of risky shift after gain and cautious shift after loss (found for a close friend in Study 1). The shapes of the underlying utility functions provide an initial account for this continuum with the magnitude of the parameters that define the functions. More research is needed to understand this continuum and to be able to predict and control the directions of the shifts.

Finally, future research is needed to better understand the effect of empathy on errors of affective forecasting for the self and for the other. Research demonstrates that empathy helps people to predict their own affective responses more accurately [51,52,53], but also demonstrates that empathetic concerns are amplified for familiar and significant others [28]. The current findings did not observe an effect of empathy on predictions for one’s own preferences and demonstrated an association between higher empathy and larger errors for a close friend. More research is needed to identify the underlying mechanisms that drive the different effects of empathy.

## 5. A Comment Regarding Practical Implications

Considering practical implications, the potential for opposite directions of affective forecasts for oneself and close others may have a critical influence on decision-making in domains such as healthcare and policymaking. For instance, the preference for drug B over drug A in the kidney cancer treatment opening story demonstrates how patients’ forecasts of their own experiences can differ from their physicians’ forecasts for them. Understanding such biases can lead to more effective decision-making processes. Recognizing and addressing self–other differences in the impact bias can improve communication and outcomes, ensuring that decisions align more closely with the actual preferences and needs of individuals and their loved ones.

## Figures and Tables

**Table 1:** The 16 gambles of the sequential gambling paradigm.

Problem No.	Values of 1st and 2nd Gambles		EV	Outcome of 1st G
	Gain	Loss		
1	200	−100	50	L
2	200	−200	0	G
3	120	−100	10	G
4	180	−200	−10	G
5	200	−120	40	G
6	180	−100	40	L
7	100	−120	−10	L
8	200	−160	20	L
9	200	−220	−10	G
10	160	−100	30	L
11	200	−180	10	L
12	200	−100	50	G
13	140	−100	20	G
14	200	−140	30	G
15	80	−100	−10	L
16	100	−100	0	L

Each gamble offers an equal chance for a gain and for a loss.

**Table 2:** Average proportions of accepting a gamble for anticipated vs. experienced outcome.

	Plans for		Revised Decisions for	
	Anticipated Gain	Anticipated Loss	Experienced Gain	Experienced Loss
Self	0.63	0.58	0.56	0.65
Other	0.68	0.47	0.44	0.65
Close Friend	0.43	0.62	0.68	0.52

**Table 4:** Average proportions of accepting a gamble for imagined vs. experienced outcome.

	Plans for		Revised Decisions for	
	Anticipated Gain	Anticipated Loss	Experienced Gain	Experienced Loss
**Self**	0.67	0.51	0.53	0.64
**Close Friend**	0.53	0.50	0.51	0.53
				
Self (Empathy Q1)	0.64	0.65	0.46	0.66
Friend (Empathy Q1)	0.53	0.44	0.44	0.61
				
Self (Empathy Q4)	0.70	0.54	0.56	0.65
Friend (Empathy Q4)	0.42	0.62	0.61	0.40

## Data Availability

The data files and statistical analyses files, as well as the syntax for the simulation of the reference-change model, can be accessed here: https://osf.io/uh3af/?view_only=aa3e1392c1a342e9bf5c0c9d17f69daf (accessed on 25 October 2024).

## Appendix A. The Reference-Change Model [35]

The reference-change model predicts the joint probabilities of the four pairs of joint choices of planned decisions and revised decisions: P(accept-accept) is the joint probability of planning to accept the gamble and then deciding to accept the gamble in the revised decision that follows experience; likewise, P(accept-reject) is the joint probability of planning to accept the gamble and then deciding to reject the gamble in the revised decision that follows experience. P(reject-accept) is the joint probability of planning to reject the gamble, and then deciding to accept the gamble in the revised decision that follows experience, and finally, P(reject-reject) is the joint probability of planning to reject the gamble and then deciding to reject the gamble in the revised decision that follows experience.

The equations are based on the marginal probabilities at *t*1 (before the experience of the gamble) *P*(Accept1), and at *t*2 (after the experience of the gamble) *P*(Accept2). It is assumed that at *t*2, a decision maker either recalls and repeats the previous choice at *t*1 or makes a separate independent choice. The probability of repeating the previous choice is represented by a parameter denoted *m*. The joint probabilities are given by Equations (A1a)–(A1d).

*P*(accept-accept) = *P*(Accept1) · [*m* + (1 − *m*)*P*(Accept2)] (A1a)

*P* (accept-reject) = *P*(Accept1) · (1 − *m*)*P*(Reject2) (A1b)

*P*(reject-accept) = *P*(Reject1) · (1 − *m*)*P*(Accept2) (A1c)

*P*(reject-reject) = *P*(Reject1) · [*m* + (1 − *m*)*P*(Reject2)] (A1d)

If *m* = 1 (i.e., the decision maker recalls and repeats the decision from *t*1), then decisions at *t*1 and *t*2 are identical and there will be no reversals (i.e., no forecast errors due to mere consistency). If *m* = 0, the decision maker does not recall the previous choice or abandons it; the two choices are independent and reversals are possible (i.e., an independent evaluation for the revised decision). When 0 < *m* < 1, there is some dependence, yet the possibility for reversals exists (i.e., consistency and the re-evaluation both affect the revised decision). To predict the joint probabilities, both m and the marginal probabilities *P*(Accept1) and *P*(Accept2) should be estimated.

A simplified version of decision field theory [35] was used to predict the marginal choice probabilities. This version of decision field theory describes choice as a process in which a preference evolves and is stabilized when it exceeds some threshold, *θ*. The preference is determined by the ratio of the valence difference (*d* = difference between the utility of the gamble and the utility of the alternative sure outcome) and the risk involved in the gamble (i.e., the gamble’s variance *σ*^2^).

According to the model, the probability of choosing the gamble over a certain outcome is given by Equation (A2):

*P*(Accept) = 1/{1 + exp[−2 · *θ* (*d*/*σ*^2^)]} (A2)

where *d* = *μ − u*(C) is the difference between the gamble’s subjective expected utility (*μ*) and the utility of the alternative certain outcome (*u*(C)). The subjective expected utility of the gamble is defined as *μ* = p·*u*(g) + (1 *−* p)·*u*(l). That is, the utilities of the gain and loss, *u*(g) and *u*(l), are weighted by their subjective probabilities, p and (1 *−* p). The variance of the gamble, *σ^2^* = p·*u*(g)^2^ + (1 − p)*u*(l)^2^ − *μ*^2^, represents the risk involved in the gamble; the larger the variance, the riskier the gamble. To determine the utilities, the model incorporates a two-part power function where the utility of a gain is *u*(g) = g*^α^* and the utility of a loss is *u*(l) = −*λ* (−l)*^α^* [48,56]. Depending on the values of *α* and *λ,* the utility function may be similar or dissimilar to a prospect-theory utility function. The abovementioned parameter *θ* represents the threshold for a stabilized preference (i.e., decision) which is an important determinant of decision time (not measured in the sequential gambling task).

A Baseline model

A baseline model is set as a benchmark for comparison for the reference-change model. In the baseline model, the marginal probabilities *P*(Accept1) and *P*(Accept2) are computed based on the assumption that at *t*1 and at *t*2, the second gamble is evaluated against a neutral reference point (i.e., *u*(C) = 0). Note that the two marginal probabilities can only differ due to m representing random fluctuations in preferences. Therefore, the probability of accept–reject or reject–accept reversals is equal. When failing to produce the observed pattern of reversals, it is of value, providing a baseline that the reference-change model must exceed.

The Reference-change model

The reference-change model is identical to the baseline model with one important exception. As before, the marginal probability *P*(Accept1) is computed based on the assumption that at *t*1, the second gamble is evaluated against a neutral reference point (i.e., *u*(C) = 0). However, the marginal probability *P*(Accept2) incorporates a reference change. At *t*2, the outcome of the first gamble shifts the reference point of the utility function as follows: Define the outcome of the first gamble as r. If the first gamble was won, the new reference point would be *u*(C) = r*^α^*. If the first gamble was lost, the new reference point would be *u*(C) = −λ (−r)*^α^*. Integrating r to the possible outcomes of the second gamble also changes its subjective expected utility (μ). The two redefined outcomes are g + r and l + r. If either of these redefined outcomes are positive, their utilities would be determined in the gain domain (i.e., if g + r > 0, *u*(g + r) = (g + r)*^α^*, if l + r > 0, *u*(l + r) = (l + r)*^α^*). If either of the redefined outcomes are negative, their utilities would be determined in the loss domain (i.e., if g + r < 0, *u*(g + r) = −*λ* (|g + r|)*^α^*, if l + r < 0, *u*(l + r) = −*λ* (|l + r|)*^α^*).

In sum, this model has the same number of free parameters (*α*, *λ*, *θ*, *m*) as the baseline model. However, it is forced to predict different probabilities for the accept–reject and reject–accept reversals. Compared to the baseline model, this feature may be an advantage or a disadvantage, depending on whether the predicted differences are in the correct direction.

Following Barkan and Busemeyer [35], model estimations were carried at the individual level, trying to reproduce for each participant the set 16 pairs of decisions (at *t*1 and *t*2) with one set of 4 parameters (2 of which determine the utility function). The parameter estimation used the *Fmins* procedure that chooses the first set of parameters that maximize the log likelihood for each participant (i.e., minimize *χ*^2^ lack of fit).

Barkan and Busemeyer [35] compared the reference-change model to the baseline model, as well as to an alternative model (not specified here) where updates that are led by experience are driven by a change in subjective probabilities rather than a change in the reference point. The reference-change model provided the best fit (i.e., minimized *χ*^2^ lack of fit).

The parameter estimation was performed with a MATLAB procedure (the syntax for the simulation of the reference-change model can be accessed here: https://osf.io/uh3af/?view_only=aa3e1392c1a342e9bf5c0c9d17f69daf, accessed on 25 October 2024).
